# Cognitive consequences of COVID-19: results of a cohort study from South America

**DOI:** 10.1590/0004-282X-ANP-2021-0320

**Published:** 2021-11-30

**Authors:** Lucía Crivelli, Ismael Calandri, Nicolás Corvalán, María Agostina Carello, Greta Keller, Carlos Martínez, Micaela Arruabarrena, Ricardo Allegri

**Affiliations:** 1 Fleni, Department of Cognitive Neurology, Buenos Aires, Argentina. Fleni Department of Cognitive Neurology Buenos Aires Argentina; 2 Universidad de la Costa, Barranquilla, Colombia. Universidad de la Costa Barranquilla Colombia

**Keywords:** COVID-19, Neuropsychology, Cognitive Dysfunction, Executive Function, Memory, COVID-19, Neuropsicología, Disfunción Cognitiva, Función Ejecutiva, Memoria

## Abstract

**Background:**

Neurological and psychiatric manifestations associated with SARS-CoV-2 infection have been reported throughout the scientific literature. However, studies on post-COVID cognitive impairment in people with no previous cognitive complaint are scarce.

**Objective:**

We aim to investigate the impact of COVID-19 on cognitive functions in adults without cognitive complaints before infection and to study cognitive dysfunction according to disease severity and cognitive risk factors.

**Methods:**

Forty-five post-COVID-19 patients and forty-five controls underwent extensive neuropsychological evaluation, which assessed cognitive domains such as memory, language, attention, executive functions, and visuospatial skills, including psychiatric symptomatology scales. Data were collected on the severity of infection, premorbid medical conditions, and functionality for activities of daily living before and after COVID-19.

**Results:**

Significant differences between groups were found in cognitive composites of memory (p=0.016, Cohen’s d= 0.73), attention (p<0.001, Cohen’s d=1.2), executive functions (p<0.001, Cohen’s d=1.4), and language (p=0.002, Cohen’s d=0.87). The change from premorbid to post-infection functioning was significantly different between severity groups (WHODAS, p=0.037). Self-reported anxiety was associated with the presence of cognitive dysfunction in COVID-19 subjects (p=0.043).

**Conclusion:**

Our results suggest that the presence of cognitive symptoms in post-COVID-19 patients may persist for months after disease remission and argue for the inclusion of cognitive assessment as a protocolized stage of the post-COVID examination. Screening measures may not be sufficient to detect cognitive dysfunction in post-COVID-19 patients.

## RESUMEN

**Antecedentes:**

Las manifestaciones neurológicas de la infección por SARS-CoV-2 han sido reportadas en la literatura científica. Sin embargo, los estudios cognitivos post COVID-19 en personas sin queja cognitiva previa son escasos.

**Objetivo:**

Nuestro objetivo es investigar el impacto cognitivo del COVID-19 en adultos sin quejas cognitivas previas a la infección y estudiar el desempeño cognitivo de acuerdo a la severidad de la enfermedad y a los factores de riesgo cognitivo.

**Métodos:**

Cuarenta y cinco pacientes post COVID-19 y cuarenta y cinco controles sanos apareados por edad, género y educación realizaron una evaluación neuropsicológica, que evalúa memoria, lenguaje, atención, funciones ejecutivas, habilidades visuoespaciales, incluyendo además escalas de sintomatología psiquiátrica, y la recopilación de datos sobre la severidad de la infección, la salud premórbida y la funcionalidad.

**Resultados:**

Se encontraron diferencias significativas entre los grupos en los compuestos cognitivos de memoria (p=0,016, d de Cohen= 0,73), atención (p<0,001, d de Cohen= 1,2), funciones ejecutivas (p<0,001, d de Cohen=1,4) y lenguaje (p=0,002, d de Cohen=0,87). El cambio del funcionamiento premórbido al funcionamiento posterior a la infección, fue significativamente diferente entre los grupos de gravedad (WHODAS; p=0,037). La ansiedad autoinformada se asoció con la presencia de disfunción cognitiva en los sujetos de COVID-19 (p=0,043).

**Conclusión:**

Nuestros resultados sugieren que los síntomas cognitivos en pacientes post COVID-19 pueden permanecer hasta tres meses después de la remisión de la enfermedad. Este estudio aboga a favor de incluir la evaluación cognitiva como una etapa protocolizada del examen post COVID-19. Es posible que las medidas de cribado no sean suficientes para detectar la disfunción cognitiva en los pacientes post COVID-19.

## INTRODUCTION

Severe acute respiratory syndrome coronavirus 2 (SARS-CoV-2) has infected over 110 million people worldwide, resulting in more than 2 million deaths globally. The predominant consequences of the virus are respiratory; however, neurological manifestations of COVID-19^1^ have been reported with a frequency of 36.4 to 84%^2^. Neurological manifestations include altered consciousness, acute neuropathies, cognitive impairment, and skeletal muscle injury. It is important to note that neurological symptoms were also found in patients who did not present with the typical signs of COVID-19 as the only manifestation^3^.

Neurological manifestations associated with SARS-CoV-2 infection usually occur during the second week of illness and are mainly observed in critically ill patients^4^. While psychiatric and neurological alterations have been consistently reported^5^^,^^6^^,^^7^, few studies currently report cognitive deficits among COVID-19 survivors^8^^,^^9^^,^^10^^,^^11^^,^^12^. These studies report cognitive deficits in mild and moderate cases of the infection^13^, with significant impairment of executive functions, memory, and attention^14^. In addition, cognitive deficits have been reported in studies performed in the acute phase of the infection^15^ and in recovered patients.

With more than 40 million confirmed cases and 1.5 million deaths, Latin America has been one of the regions most affected by the pandemic^16^. However, evidence of post-COVID cognitive impairment in the region is scarce. Neurological alterations in post-COVID-19 patients were reported in a cohort study of 63 hospitalized post-COVID patients^17^. The most frequent neurological complications were ischemic stroke in 30 patients (47.6%) and encephalopathy in 17 patients (27%), followed by seizures, hemorrhagic stroke, and headache. These studies highlight the need to evaluate cognitive symptoms after the disease to assess patients' cognitive status and design rehabilitation strategies.

Regarding the study of post-COVID cognitive impairment in the Latin American region, Del Brutto^13^ compared cognitive change (decline) in a cohort of middle-aged and older adults from a community in Atahualpa. The study compared changes between pre-pandemic measures and post-pandemic assessment and between seropositive and seronegative individuals. Results show a 21% cognitive decline in the sample of COVID-19 survivors versus only 2% of the seronegative group. This study, as well as others^18^^,^^19^^,^^20^, assessed cognitive dysfunction using cognitive screening tools such as the Montreal Cognitive Assessment (MoCA), Mini-Mental State Examination (MMSE), and Telephone Interview for Cognitive Status (TICS). 

The aim of this study was to describe the cognitive profile of a cohort of COVID-19 survivors that attended a neurological clinic in Buenos Aires, Argentina. In addition, the impact of COVID-19 on cognitive functions in adults without cognitive complaints before infection, cognitive dysfunction according to disease severity, and cognitive risk factors were evaluated.

## METHODS

### Study design and participants

We report data from 45 post-COVID-19 patients recruited from an outpatient neurological clinic by attending neurologists. Patients were evaluated for an average of 142 days after illness. Inclusion criteria were: a positive SARS-CoV2 RT-PCR result from nasopharyngeal swabs, age > 18 years, and no pre-infection cognitive complaint. Exclusion criteria were: significant upper limb impairment, visual acuity or visual field deficits, drug use, or psychiatric disorders. The local ethics committee approved the protocol and all subjects signed an informed consent form before assessments.

Neuropsychological assessment was performed using an extensive cognitive battery. Additionally, data were collected on the characteristics of the acute COVID-19 episode, premorbid medical conditions, and functionality for activities of daily living (ADL) pre-and-post COVID-19. Disease severity was classified as asymptomatic, mild, moderate, and severe according to WHO^21^. The study was designed in three steps to answer three specific questions: 1) Are there cognitive deficits in post-COVID-19 subjects with a cognitive complaint? 2) Is it possible to describe a cognitive profile for these patients? 3) Are there identifiable risk factors for the occurrence cognitive complaints? First, subjects were compared with forty-five healthy control (H.C.) subjects with no history of SARS-CoV-2 infection matched for sex, age, and educational level. In a second instance of the study, we created cognitive composites to better understand cognition in the domains. The development of a comprehensive cognitive battery for post-COVID patients took several months. For this reason, some patients did not receive the complete assessment regardless of their cognitive performance. To avoid sample bias, only subjects with complete data (N=29) and their matched controls were included. Finally, in the third instance, a risk model was constructed by logistic regression for the clinical and demographic variables evaluated in the study.

### Outcomes

Participants underwent a comprehensive neuropsychological evaluation that included anxiety, depression, and functionality scales. In addition, assessment of clinical illness characteristics and risk factors were self-reported using structured questionnaires and scales. 

### Cognitive assessment

Cognitive screening was performed using the Argentine version of the MoCA^22^.^ ^

The extensive neuropsychological assessment included attention, memory, language, executive functions, and visuospatial skills tests. Attention was assessed with Trail Making A^23^, Digit Span Forwards^24^, and Digit-Symbol Coding^25^. Memory was tested using Craft Story 21^26^, Rey Auditory Verbal Learning Test^27^, and Delayed Recall from the Benson Figure Test^28^. Executive function assessment included Trail Making B^23^, Wisconsin Card Sorting Test^29^, Stroop Test^30^, and phonological fluency^31^. Benson Figure and Clock Drawing Test^32^ assessed visuospatial skills. Finally, the language was assessed using the Multilingual Naming Test^33^ and semantic fluency^34^.

### Neuropsychiatric and functional assessment

The Hospital Anxiety and Depression Scale^35^ was administered for neuropsychiatric screening purposes. In addition, WHODAS 2.0 Functional Scale^21^ was used to assess pre-and post-COVID-19 changes in ADL. Subjects were instructed to answer the questions regarding their pre-infectious status.

### Disease severity and risk factors for cognitive impairment assessment

Participants completed the CAIDE (Cardiovascular Risk Factors, Aging, and Incidence of Dementia) Dementia Risk Score, a test for estimating risk of dementia in the general population^36^. This test combines self-reported measures of age, education, sex, hypertension, body mass index, hypercholesterinemia, and physical activity into a dementia risk score. In addition, we assessed risk factors for COVID disease severity such as diabetes, smoking, cardiac disease, and chronic obstructive pulmonary disease (COPD) using an ad hoc yes/no binary questionnaire.

### Statistical analysis

All variables were tested graphically and analytically for normality assumptions. For variables with normal distribution, summary statistics are presented as mean and standard deviation (S.D.), and for variables without normal distribution, median (M) and interquartile range (IQR) are used. Group differences were evaluated using independent t-tests, one-way ANOVA, and the Mann-Whitney U or Kruskal-Wallis test was used according to data distribution. To prevent an increase in the family-wise error rate (FWER) associated with multiple independent hypothesis testing, we used the Benjamini Hochberg procedure when more than four independent variables were analyzed in one group. We also approached the FWER problem with the creation of composites (see next section).

A risk model for cognitive impairment diagnosis was built. In this cohort, cognitive impairment diagnosis was defined as a Z score of -1.5 or less in at least one cognitive composite. A logistic regression model was used to assess this risk. Results are presented as odds ratios (OR) for every risk factor and accuracy model through Akaike information criteria (AIC). For all tests, a significance level was set at p<0.05.

Results from neuropsychological tests were calculated using composite scores. The composite quantifies cognitive function across multiple tests with greater statistical power than individual measures. It consolidates type 1 error into a single outcome. Domain-specific composites were constructed using the tests from the neuropsychological battery that better predicted cognitive impairment for each cognitive domain. 

The domain-specific composite outcome was calculated as follows:

- Scores for each contributing test were converted to Z scores according to normative data. When necessary, Z scores were corrected so that positive scores reflected better performance and negative scores reflected worse performance;
- Memory Composite: RAVLT learning score and RAVLT Delayed Score, Benson Figure Test Delayed score;
- Attention Composite: TMT A, Digit span Forwards;
- Executive Composite: TMT B, Digit span Backward, Phonological fluency;
- Language Composite: MINT score, Semantic fluency.

## RESULTS

### Demographic results

The characteristics of the complete cohort of 45 post-COVID-19 patients (M = 50) and 45 H.C. matched by age, sex, and education are shown in Table 1. There were no differences between patients and H.C. in the estimated risk for dementia as measured by the CAIDE Score (p=0.3).

**Table 1.** Demographic results.

Characteristic	Healthy controls (N = 45)	Post-COVID (N = 45)	p-value^2^
Age (y)	57 (46, 64)	50 (43, 63)	0.4
Sex: female	20 (44%)	22 (49%)	0.7
Education (y)	17.00 (15.00, 18.00)	17.00 (15.00, 18.00)	>0.9
Duration of infection (days)	-	15.5 (2.2)	
Evaluation post-infection (days)	-	142 (75.9)	
Hospitalization	-	14 (31%)	
CAIDE dementia score	6.00 (3.00, 7.00)	5.00 (2.00, 7.00)	0.3

Data are reported as median (interquartile range), n (%), or median (standard deviation); ^2^Wilcoxon rank sum test or Pearson's Chi-squared test; CAIDE: Cardiovascular Risk Factors, Aging, and Incidence of Dementia Dementia Risk Score.

### General cognitive performance and neuropsychiatric symptomatology

When comparing H.C. and patients on individual test measures (Table 2), no significant differences were found in the complete sample for the screening measures (MoCA p=0.15; MMSE p=0.4). However, significant differences were found between groups in all of the memory and attention scales. Language measures were significantly lower for semantic and phonological fluency in the patient group, but not for naming. Significant differences in executive performance were found between groups, with a better performance of H.C. in alternating attention, categorization, and perseverations. No differences were found in the visuospatial domain in copying complex figures; however, significantly lower performance was observed in the patient group for the CDT. It is important to note that this test does not exclusively assesses visuospatial abilities but also includes semantic and executive components. The neuropsychiatric variables of depression and anxiety did not differ between groups.

**Table 2.** General cognitive results.

		Healthy controls (N = 45^1^)	Post-COVID (N = 45^1^)	p-value^2^
Screening	MoCA total	27.22 (1.99)	26.49 (2.90)	0.4
	MMSE/MoCA crosswalk	27.22 (1.99)	26.04 (3.33)	0.15
	CDT	9.78 (0.59)	9.13 (1.35)	0.007
Memory	RAVLT Total	50 (9)	43 (13)	0.018
	RAVLT delayed recall	10.2 (2.9)	8.2 (3.5)	0.007
	Benson figure delayed recall	12.27 (2.65)	10.50 (3.25)	0.009
Language	MINT/BNT crosswalk	30.31 (1.55)	29.47 (2.12)	0.058
	Semantic fluency	22.6 (4.5)	18.9 (5.2)	<0.001
	Phonological fluency (p)	17.9 (4.3)	14.1 (4.6)	<0.001
Attention	Digit span (direct)	6. 89 (0.93)	5.89 (1.30)	<0.001
	Digit span (indirect)	5.09 (0.95)	4.04 (1.19)	<0.001
	Trail making test A	29 (7)	47 (25)	<0.001
	WAIS-IV Coding	13.5 (2.9)	11.8 (3.7)	0.010
Executive system	Trail making test B	62 (22)	107 (76)	<0.001
	WCST cat	6.00 (0.00)	5.59 (1.04)	0.014
	WCST pers	0.89 (1.29)	2.94 (4.63)	0.010
Visuospatial	Benson figure copy	16.24 (0.98)	16.26 (3.10)	0.2
Neuropsychiatric	HADS anxiety	8.8 (3.4)	8.5 (3.4)	0.8
	HADS depression	6.1 (3.7)	5.9 (3.5)	0.7

^1^Data are reported as mean (standard deviation); ^2^ Wilcoxon rank-sum test; MoCA: Montreal Cognitive Assessment; CDT: Clock Drawing Test; RAVLT: Rey Auditory Verbal Learning Test; MINT/BNT crosswalk: Multilingual Naming Test / Boston Naming Test crosswalk; WAIS IV: Wechsler Adult Intelligence Scale IV; WCST: Wisconsin Card Sorting Test; HADS: Hospital Anxiety and Depression Scale.

### Cognitive results by domain, severity, and impact on functionality

The complete cognitive battery including memory, attention, language and executive composite scores, neuropsychiatric, functional, and risk factor assessment was administered to a subsample of 29 patients and 29 controls.

Results from composite scores show deficits in memory (p=0.016, d= 0.73), attention (p<0.001, d=1.2), executive functions (p<0.001, d=1.4), and language (p=0.002, d=0.87). Cohen’s D was calculated for each composite to measure effect size. Effects for executive functions, attention, and language were large and effects for memory were intermediate (Table 3).

**Table 3.** Results of cognitive composites.

	Healthy control (N = 29^1^)	Post-COVID (N = 29^1^)	p-value^2^	Effect size^3^
Memory (composite)	0.20 (-0.19, 0.60)	-0.19 (-0.76, 0.06)	0.016	0.734
Attention (composite)	-0.12 (-0.57, 0.28)	-1.16 (-1.66, -0.60)	<0.001	1.272
Executive (composite)	0.10 (-0.02, 0.31)	-0.62 (-1.52, -0.21)	<0.001	1.483
Language (composite)	0.05 (-0.22, 0.42)	-0.49 (-0.76, 0.04)	0.002	0.877

^1^Data are reported as median (interquartile range); ^2^Wilcoxon rank sum test; ^3^Cohen's D.

**Figure 1.**
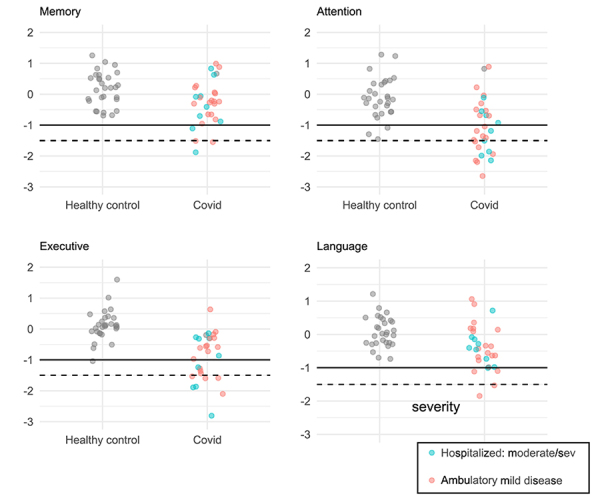
Cognitive domains across illness severity levels in in healthy controls.

The patient group was divided according to illness severity using the WHO severity scale (Table 4 and Figure 1). Results show no significant differences between the different cognitive composites across the different severity groups.

**Table 4.** Cognitive domain performance across different COVID-19 severity levels.

	Ambulatory: mild disease (N = 19^1^)	Hospitalized: moderate and severe disease (N = 9^1^)	p-value^2^
Memory (composite)	-0.19 (-0.69, -0.01)	-0.35 (-0.98, 0.06)	0.8
Attention (composite)	-1.16 (-1.60, -0.63)	-1.31 (-1.90, -0.72)	0.6
Executive (composite)	-0.62 (-1.50, -0.32)	-0.74 (-1.87, -0.21)	0.8
Language (composite)	-0.54 (-0.71, 0.11)	-0.40 (-0.76, -0.26)	>0.9

^1^Data are reported as median (interquartile range); ^2^Kruskal-Wallis rank sum test.

The change in functionality was assessed with the WHODAS 2.0, which inquires on different aspects of functioning before and after SARS-CoV-2 infection. The total score was significantly different between severity groups (p=0.011), indicating a change in functionality post-SARS-CoV-2 infection according to disease severity. In addition, WHODAS subscore results show a differential impact of cognitive functionality, social participation, and mobility between disease severity levels (p=0.007).

### Predictors of cognitive impairment

A logistical regression model was built to identify predictors of cognitive impairment among post-COVID-19 subjects. The model with the best predictive performance (AIC: 38.3) did not identify common clinical risk factors as predictors of post-COVID-19 cognitive impairment. In addition, only self-reported anxiety measured by the Hospital Anxiety and Depression Scale (HADS) showed significant results (Table 5).

**Table 5.** Predictors of cognitive impairment.

Characteristic	OR^1^	95% CI^1^	p-value
CAIDE dementia score	0.21	0.00, 1.15	0.14
Age (y)	1.19	0.89, 1.75	0.3
Sex			
Male	49.7	0.66, 41986.3	0.15
Female	49.8	0.67, 6.54	0.14
Education (y)	1.00	0.26, 6.54	>0.9
Obesity	4472.3	0.97, 5012838000	0.11
Hypertension	0.00	NA, 14383040000	>0.9
HADS Depression	2.22	1.17, 6.54	0.056
HADS Anxiety	0.49	0.18, 0.84	0.043
Physical activity	0.21	0.00, 10.1	0.4

^1^Data are reported as odds ratio (OR) and 95% confidence interval (95%CI); CAIDE: Cardiovascular Risk Factors, Aging, and Incidence of Dementia -Dementia Risk Score; HADS: Hospital Anxiety and Depression Scale. NA: Not available.

## DISCUSSION

The SARS-CoV2 infection produces multiple organ failure leading to a wide variety of symptomatology and respiratory syndromes. Neurological manifestations are frequent^2^ and diverse, and may include cognitive impairment^12^. This study focused on the description of cognitive performance in previously healthy adults with no history of cognitive impairment. Our results advocate for the importance of including cognitive assessment as a protocolized stage of post-COVID examination.

We studied patients that attended a Neurological Clinic with a post-COVID cognitive complaint. Our results show that deficits can be identified predominantly in executive functions and attention, and have a smaller effect on memory and language. Furthermore, these deficits do not vary according to disease severity as measured by the WHO's COVID-19 severity scale^37^. Notwithstanding, self-reported cognitive functionality, social participation, and mobility differ according to disease severity, indicating that the impact of cognitive impairment is higher when illness severity is increased. 

Regarding the affected cognitive domains, our results are consistent with other studies that found a similar cognitive profile, with impaired attention and executive functions in patients one to six months after infection, with severity ranging from asymptomatic^18^ to moderate and severe^10^^,^^15^^,^^38^. Furthermore, these deficits are reported in the acute phase of the disease^39^^,^^40^ to up to 6 months after recovery^13^.

Our study reported that cognitive dysfunction did not vary significantly between the mild and moderate severity groups. However, this result must be interpreted with caution because our sample size was small and, most importantly, unevenly distributed across severity levels. Most of the patients were classified as mild (n=19), and very few patients as moderate COVID-19 (N=9). Conflicting results have been found in other studies that looked into the correlation between cognition and cognitive performance. A group of studies found no association between the severity of COVID-19 and cognitive functioning^12^^,^^20^. In contrast, others found that global cognitive impairment and executive dysfunction correlated with the severity of respiratory symptoms and poorer pulmonary function^15^. Our study complements previous research but is not conclusive enough to draw conclusions. Prospective studies specifically designed to study cognitive performance and cognitive profile across different disease severity groups are needed.

Regarding possible predictors of cognitive impairment, we found no influence of commonly studied risk factors for dementia (diabetes, smoking, age, and education) nor the well-known risk factors for severe COVID-19 respiratory illness. Our study, however, found that anxiety was a predictive factor of cognitive impairment. It is a well-known that mood disorders, including anxiety, can cause cognitive dysfunction^41^. However, our study did not find significant differences between groups in anxiety or depression, and levels of anxiety of our cohort fell into the normal range in both groups. Therefore, the results must be regarded with caution.

A possible understanding of COVID-19 cognitive impairment may be based on records of other coronaviruses (such as SARS and MERS), in which long-term neuropsychological deficits were registered^42^. In these outbreaks, cognitive deficits were also centered on attention, executive functions, and memory. Furthermore, cognitive dysfunction in patients with a viral infection such as Human Immunodeficiency Virus (HIV) has also been reported in prior studies, in which deficits in attention, learning, and memory functions were reported^43^. Additionally, the Zika virus has also been reported to leave long-term cognitive sequelae^44^. The COVID-19 pandemic has brought to our attention the need to better understand the role of viral infections in cognitive impairment.

Finally, our results suggest that cognitive symptoms may be expected in patients after COVID-19 recovery and may last months after illness remission. Thus, our results underscore the need to include a cognitive assessment in post COVID-19 follow-ups to guide possible cognitive rehabilitation treatment.

Limitations of our study include heterogeneity at the time of evaluation, small sample size, and unequal frequency of disease severity levels, as well as the non-inclusion of patients with severe disease. Future studies should include a systematic analysis with larger patient cohorts and long-term follow-up. Another limitation of our study is the absence of a cognitive assessment before infection, which would allow a more accurate estimation of the impact of COVID-19 on cognitive functions. In addition, the subjects included in the study had requested a medical consultation, which could result in a population with elevated anxiety symptoms that could affect cognitive performance. Finally, a considerable number of subjects did not fully complete the cognitive assessment battery, so composites were generated to avoid sample bias between groups.

Results indicated that commonly used screening methods in the elderly population (MMSE, MoCA) are not sensitive enough to detect cognitive impairment in post-COVID patients. A more exhaustive neuropsychological examination is needed. Our study is among the first in our region to use a broad and robust neuropsychological battery, which is sensitive for detecting cognitive dysfunction post-COVID-19. The neuropsychological tests included in our composites could be recommended as an adequate neuropsychological battery for the Latin American population.
